# National Board of Health

**Published:** 1879-06-20

**Authors:** 


### *NATIONAL BOARD OF HEALTH.*

The National Board of Health closed its session in Atlanta on the 8th of May. The Sanitary Council of Mississippi held a meeting the previous day, and submitted to the Board, through Dr. Ranch the Secretary, the propositions adopted by them. The propositions being adopted, secured co-operation between the two Boards. Information relative to sanitary matters is to be gathered from all sources, and laid before a future meeting of the National Board of Health, to be held at Nashville, Tenn., on the 8th of November next.

